# Combined p14ARF and Interferon-β Gene Transfer to the Human Melanoma Cell Line SK-MEL-147 Promotes Oncolysis and Immune Activation

**DOI:** 10.3389/fimmu.2020.576658

**Published:** 2020-10-22

**Authors:** Otto Luiz Dutra Cerqueira, Maria Alejandra Clavijo-Salomon, Elaine Cristina Cardoso, Tharcisio Citrangulo Tortelli Junior, Samir Andrade Mendonça, José Alexandre M. Barbuto, Bryan E. Strauss

**Affiliations:** ^1^ Centro de Investigação Translacional em Oncologia (CTO), Instituto do Câncer do Estado de São Paulo (ICESP), Faculdade de Medicina da Universidade de São Paulo (FMUSP), São Paulo, Brazil; ^2^ Departamento de Imunologia, Instituto de Ciências Biomédicas, Universidade de São Paulo, São Paulo, Brazil; ^3^ Department of Pediatrics, Faculdade de Medicina da Universidade de São Paulo (FMUSP), São Paulo, Brazil; ^4^ Laboratory of Medical Investigation in Pathogenesis and Targeted Therapy in Onco-Immuno-Hematology (LIM-31), Department of Hematology, Hospital das Clínicas HCFMUSP, Faculdade de Medicina, Universidade de Sao Paulo, Sao Paulo, Brazil

**Keywords:** melanoma, adenovirus (Ad) vector, oncolysis, immunogenic cell death (ICD), immunotherapy

## Abstract

Immune evasion is an important cancer hallmark and the understanding of its mechanisms has generated successful therapeutic approaches. Induction of immunogenic cell death (ICD) is expected to attract immune cell populations that promote innate and adaptive immune responses. Here, we present a critical advance for our adenovirus-mediated gene therapy approach, where the combined p14ARF and human interferon-β (IFNβ) gene transfer to human melanoma cells led to oncolysis, ICD and subsequent activation of immune cells. Our results indicate that IFNβ alone or in combination with p14ARF was able to induce massive cell death in the human melanoma cell line SK-MEL-147, though caspase 3/7 activation was not essential. *In situ* gene therapy of s.c. SK-MEL-147 tumors in Nod-Scid mice revealed inhibition of tumor growth and increased survival in response to IFNβ alone or in combination with p14ARF. Emission of critical markers of ICD (exposition of calreticulin, secretion of ATP and IFNβ) was stronger when cells were treated with combined p14ARF and IFNβ gene transfer. Co-culture of previously transduced SK-MEL-147 cells with monocyte-derived dendritic cells (Mo-DCs) derived from healthy donors resulted in increased levels of activation markers HLA-DR, CD80, and CD86. Activated Mo-DCs were able to prime autologous and allogeneic T cells, resulting in increased secretion of IFNγ, TNF-α, and IL-10. Preliminary data showed that T cells primed by Mo-DCs activated with p14ARF+IFNβ-transduced SK-MEL-147 cells were able to induce the loss of viability of fresh non-transduced SK-MEL-147 cells, suggesting the induction of a specific cytotoxic population that recognized and killed SK-MEL-147 cells. Collectively, our results indicate that p14ARF and IFNβ delivered by our adenoviral system induced oncolysis in human melanoma cells accompanied by adaptive immune response activation and regulation.

## Introduction

Suppression of the immune system during tumor progression and spread is an important cancer hallmark (1). Tumor cells actively evade recognition and destruction by both innate and adaptive branches of the immune system (2, 3). Acquisition of defects in anti-viral pathways in tumor cells, such as those mediated by interferons (IFNs), contributes to immune evasion (4, 5). This opens important perspectives for immunotherapies that seek to restore anti-tumor immune responses. Oncolysis, the rupture of malignant cells in response to a therapeutic modality, represents an important first step. However, to be effective, induction of oncolysis alone would have to reach essentially all tumor cells. Instead, oncolysis that is accompanied by immune activation would be expected to provide a broader effect at the site of treatment and may even provide a systemic benefit. This has been seen in therapy with oncolytic viruses, though combination with other immunotherapies may be required in order to achieve elimination of non-treated tumor foci (6, 7).

There are different strategies for inducing oncolysis, yet cell death, such as by apoptosis, may not elicit an immune response and may even lead to tolerance (8). In contrast, cell death accompanied by the release of immunogenic cell death (ICD) factors serves to alarm the immune system by attracting and activating immune cell populations (9–11). To this end, virus-mediated oncolysis, such as that seen with oncolytic virotherapy, promotes cell death along with the liberation of pathogen-associated molecular patterns (PAMPs), damage-associated molecular patterns (DAMPs), as well as tumor antigens that together can cooperate with the activation of dendritic cells which, in turn, prime tumor-specific T cells (5, 12–14). Viruses that trigger oncolysis have revealed strong potential as immunotherapeutic approaches (14, 15) with many clinical trials currently recruiting (as per clinicaltrials.gov), as well as FDA approval for use in patients (16, 17).

Our group has been working on the development of non-replicating adenoviral vectors that induce oncolysis due to the combined activity of the p14ARF and IFNβ transgenes as well as antiviral response (18). While p14ARF (alternate reading frame of the CDKN2a locus, p19Arf in mice, p14ARF in humans) acts as the functional partner of p53, IFNβ is a critical cytokine that contributes to innate and adaptive anti-tumor responses (19). Using mouse models, our data indicate that transgene activity plus an antiviral response culminate in ICD, thus our approach may be considered as a strategy for cancer immunotherapy (20–22). However, IFNβ acts in a species specific manner (23), thus the phenomena seen in mouse models must be verified using human cell lines.

Melanoma is a highly lethal neoplasm due to the difficulty in treatment after metastatic spread (24, 25). Peculiarities in the melanoma genotype shaped the rationale of the adenoviral strategy used in this work. Since 80% of melanomas preserve p53 in its wild-type form (26), we have incorporated a p53-responsive promoter (PG) to drive transgene expression (18). The p14ARF transgene is expected to activate endogenous p53, in turn providing both tumor suppressor function and transactivation from the PG promoter (27). In addition to its role in immune activation, the IFNβ pathway is also known to promote cell death *via* the p53/p14ARF axis (28, 29). Besides that, deletions are commonly found in the chromosome 9p21 gene cluster where CDKN2a, p14ARF, and IFNβ are located (30–33), reinforcing the importance of the p14ARF and IFNβ transgene combination.

Here, we show a critical advance in the development of our approach since we explore combined p14ARF and IFNβ gene transfer in a human melanoma cell line, SK-MEL-147. We confirmed oncolysis and also reveal that combined gene transfer is required for the induction of ICD, characterized by emission of DAMPS, activation of dendritic cells from healthy donors and their ability to prime T cells to, then, carry out tumor cell cytolysis. Thus, we suggest that the oncolysis and subsequent activation of immune functions predict that our adenovirus-mediated p14ARF plus IFNβ gene transfer approach could act as an immunotherapy in humans.

## Material and Methods

### Cell Lines

The SK-MEL-147 human melanoma cell line was authenticated by analysis of short tandem repeats using GenePrint 10 (Promega, Internal Standard-ILS 600, performed by the Rede Premium Core Facility, FMUSP) and tested negative for mycoplasma by a PCR assay using conditioned medium as template and amplification using the following oligonucleotides:

Myco F: 5’-GGG AGC AAA CAC GAT TAG ATA CCC T -3’
Myco R: 5’-TGC ATT ATC TGT CAC TCT GTT AAC CTC -3’

This cell line as well as HEK293 were cultured in DMEM with 10% fetal calf serum, supplemented with antibiotic-antimycotic (Thermo Fisher Scientific, Waltham, MA, USA) and maintained at 37°C and 5% CO_2_ atmosphere.

### Construction, Production, and Titration of Adenoviral Vectors

The strategy for constructing the adenoviral vectors has been described previously (21).

For the generation of the recombinant adenovirus we first constructed the “pEntr-PG” vector containing transgenes of interest: i) Luc2, used as control, ii) Luc2-p14ARF, and iii) Luc2-hIFNβ (**Figure S1**). Next, site directed recombination was performed with the “destiny” vector encoding the Ad5 backbone (non-replicating, E1/E3 deleted, RGD modified fiber) utilizing Gateway L/R Clonase II Enzyme (Life Technologies, Carlsbad, CA, USA) as previously described (21, 34), giving rise to AdRGD-PG-Luc2, AdRGD-PG-Luc2-p14ARF, and AdRGD-PG-Luc2-hIFNβ. Following viral amplification, purification was performed using an iodixanol gradient followed by desalting, as described by Peng et al. (35) and as per our previous studies (21, 36). For the determination of biological titer, we used the Adeno-X Rapid Titer Kit (Clontech, Mountain View, CA, USA) which is based on immunodetection of the adenoviral hexon protein in transduced cells. The biological titer (transducing units per milliliter, TU/ml) was used for the calculation of the multiplicity of infection (MOI) indicated in each experiment.

### Cell Transduction

SK-MEL-147 cells were seeded in medium containing 2% FBS together with the corresponding vectors at a final MOI of 50: i) AdRGD-PG-Luc2, ii) AdRGD-PG-Luc2-p14ARF, and iii) AdRGD-PG-Luc2-hIFNβ and iv) combination of AdRGD-PG-Luc2-p14ARF and AdRGD-PG-Luc2-hIFNβ (p14ARF+IFNβ, MOI 25 each). After 4 h incubation at 37°C, an equal volume of DMEM/10% FBS was added and the cells incubated for 24 and 48 h, unless otherwise noted. The described strategy was employed to guarantee the consistent number of viral particles for each treatment condition.

### Transgene Expression

For validation of luciferase transgene activity, we used the Luciferase Assay System kit as per the manufacturer’s protocol (Promega, Madison, WI, USA). Sample luminescence was read on a 1420 Multilabel Counter VICTOR3 (Perkin Elmer, Waltham, MA, USA). The luminescence results were normalized by protein concentration. Interferon-β detection was performed using the Verikine human IFN beta ELISA kit (PBL Assay Science, Piscataway, NJ, USA) and conditioned medium derived from transduced cell cultures. For p14ARF, we use immunofluorescence and Western blot as previously described (37–39), using anti-p14ARF antibody (Santa Cruz Biotechnology, Dallas, TX, USA). Immunofluorescence detection was obtained using EVOS FL Cell Imaging System (Thermo Fisher Scientific).

### Evaluation of Cell Death by Flow Cytometry

Cells were transduced and incubated as described before harvest of both detached and adherent cells, fixation in 70% ethanol, RNAse treatment and staining with PI (21). Alternatively, fresh cells were treated with the LIVE/DEAD ™ Fixable Green Dead Cell Stain Kit, for 488 nm excitation (Thermo Fisher Scientific, #L23101) following the manufacturer’s recommendations. For either assay, cells were subjected to flow cytometry and analysis using the manufacturer’s software (Attune™, Thermo Fisher Scientific). For measurement of caspase activity, transduced cells were collected and treated with Cell Event Caspase 3/7 Green Detection reagent (Thermo Fisher Scientific, Cat. No. C10423) diluted and incubated according to the recommendations of the manufacturer. The proportion of fluorescent cells resulting from activation of caspases 3 and 7 was assessed by flow cytometry (Attune™, Thermo Fisher Scientific).

### *In Situ* Gene Therapy

All animal experimentation and the protocols described here were approved by the Ethics in Animal Use Committee (FMUSP) and performed at the Centro de Medicina Nuclear (CMN), FMUSP, São Paulo, Brazil. Assays were performed using 8–12 weeks old female Nod-Scid mice obtained from the Bioterio Central, FMUSP. After local trichotomy, SK-MEL-147 cells (1 × 10^6^) were implanted subcutaneously in the left flank and animals were observed until tumors reached 60 mm^3^ and treatment was initiated. Intratumoral injection was performed on four occasions at 48-h intervals (days 1, 3, 5, and 7), applying 2 × 10^8^ TU in 50 µl of 1× PBS as excipient. Tumors were treated with PBS, AdRGD-PG-Luc2 or AdRGD-PG-Luc2-IFNβ, AdRGD-PG-Luc2-p14ARF, and combination of p14ARF+IFNβ. The number of animals/group is indicated in the figure legends. Tumors were measured with calipers and the volume calculated according to the formula: ½ × (length) × (width)^2^ (39, 40). We consider the tumor volume of 1000 mm^3^ as experimental end point when the animals were anesthetized in a chamber with 4% isoflurane and euthanized by CO_2_ inhalation. Otherwise, animals that did not reach the end point were monitored until 60 days. Distribution of animals/cage was maintained according to the pre-defined experimental groups at the beginning of the treatment. Alternatively, on experimental day 9 (48 h after the last treatment), some of these animals were euthanized and samples collected for histologic analyses.

### Immunogenic Cell Death Assays

The ICD markers were analyzed following the protocols previously described (21). For each assay, 1 × 10^5^ cells were treated with the vectors for 48 h, and then, cells and supernatant were collected. For the evaluation of calreticulin exposure through flow cytometry, cells were probed with rabbit anti-calreticulin antibody (Novus Biologicals, Littleton, CO, USA), followed by the Alexa488-conjugated anti-rabbit secondary antibody (Thermo Fisher Scientific). The cells were then analyzed using the Attune cytometer (Thermo Fisher Scientific). The ATP secreted in the media was evaluated using the ENLITEN ATP Assay System (Promega), following the manufacturer protocol, and the luminescence obtained with tube reader Sirius L Tube Luminometer (Titertek Berthold, Germany).

### Isolation of Peripheral Blood Mononuclear Cells (PBMCs), Mo-DCs Differentiation, and Activation With Transduced Tumor Cells

All experiments were performed after the approval of the institutional Committee for Ethics in Research (Fundação Pró-Sangue, CEP#03, FMUSP). Healthy donors’ peripheral blood was obtained from leukoreduction chambers (41) after signed written informed consents. PBMCs were isolated by density gradient centrifugation over Ficoll-Paque (GE Healthcare, Uppsala, Sweden). For the generation of monocyte-derived DCs, PBMCs were either plated for 2 h for adherence of monocytes to the plastic and subsequent removal of non-adherent cells (42) or by positive magnetic selection (Milteny Biotec, Bergisch Gladbach, Germany) for the isolation of CD14^+^ monocytes. The monocytes were cultured at 37°C and 5% CO_2_ in RPMI-1640 medium supplemented with 10% FBS, antibiotic-antimycotic (Thermo Fisher Scientific) and 50 ng/ml of IL-4 plus 50 ng/ml of GM-CSF (both from PeproTech, Rocky Hill, NJ, USA) for 5 days to obtain immature monocyte-derived dendritic cells (iDCs) (43). At day 5, iDCs cells were harvested, washed, counted, and activated for 24 h with previously transduced SK-MEL-147 cells at a 1:1 and 1:10 ratios. Cells were harvested and analyzed by flow cytometry using CD209 (DCN46, #551545, BD), HLA-DR (G46-6, #556643, BD), CD80 (L307.4, #340294, BD), CD83 (HB15e, #561132, BD), CD86 (2331, #561124, BD) or used for the priming of autologous T cells.

### T Cell Isolation and Priming

T cells were enriched by either recovery of non-adherent cells after 2 h of PBMCs plastic adherence or by negative magnetic selection with the Pan T cell isolation kit (Milteny Biotec). Aliquots of T cells were cryopreserved until used in subsequent experiments. For T cell priming, activated Mo-DCs were harvest, washed, counted, and co-cultured for 7 days with autologous T cells at a 1:10 ratio in 96-well U-bottom plates (Corning, Tewksbury, MA, USA), with human IL-7 (50ng/ml), IL-2 (250 U/ml), and IL-15 (5 ng/ml) (all from Peprotech) with media replacement with fresh cytokines every 3 days. T cell proliferation was determined by carboxyfluorescein succinimidyl ester (CellTrace™ CFSE Cell Proliferation Kit, #C34554, Thermo Fisher Scientific) dilution, where T cells were previously stained with 5 μM CFSE. After 7 days, T cells were harvested and stained for surface markers CD3 (SK7, #340542, BD), PD1 (EH12.1, #561273, BD), TIM3 (F38-2E2, #25-3109-42, Thermo Fisher Scientific), LAG3 (3DS223H, #12-2239-41, Thermo Fisher Scientific), and CFSE dilution was determined by flow cytometry.

### Cytolytic T Cell Activity

Primed T cells were seeded in 96-well U-bottom plates together with fresh non-transduced SK-MEL-147 in a 10: 1 ratio. After 48 h of incubation, SK-MEL-147 viability was assessed through LIVE/DEAD staining (Thermo Fisher Scientific) by flow cytometry. SK-MEL-147 cells were gated based on size (FSC^hi^) and absence of CD3 staining.

### Cytokine Measurements by CBA

To determine the cytokine profiles of stimulated T cells, we use co-cultures with allogeneic Mo-DCs primed with adenoviral-transduced SK-MEL-147 cells. Supernatants were collected after 3 days of cultures and analyzed by CBA. Analysis of IL-2, IL-4, IL-6, IL-10, IL-17A, TNF-α, and IFN-γ levels was performed using the Cytokine Bead Array Th1/Th2/Th17 kit (BD Biosciences, San Jose, CA, USA). Samples were acquired with a FACS II LRS Fortessa (Becton, Dickinson and Company, USA) according to the manufacturer’s instructions and analyzed using the FCAP Array Software 3.0 (BD Biosciences, San Jose, CA, USA).

### Statistical Analysis

Statistical analyses were performed using GraphPad Prism software version 5.0 (GraphPad Software, San Diego, CA, USA). Statistical significance was calculated using one-way or two-way ANOVA when appropriate, followed by Bonferroni post-test. Differences were considered significant when p < 0.05. Number of replicates and sample sizes for all experiments are detailed in the figure legends.

## Results

### Adenovirus-Mediated Gene Transfer of p14ARF and IFNβ Induces Cell Death in Human Melanoma Cells

**Figure 1:**
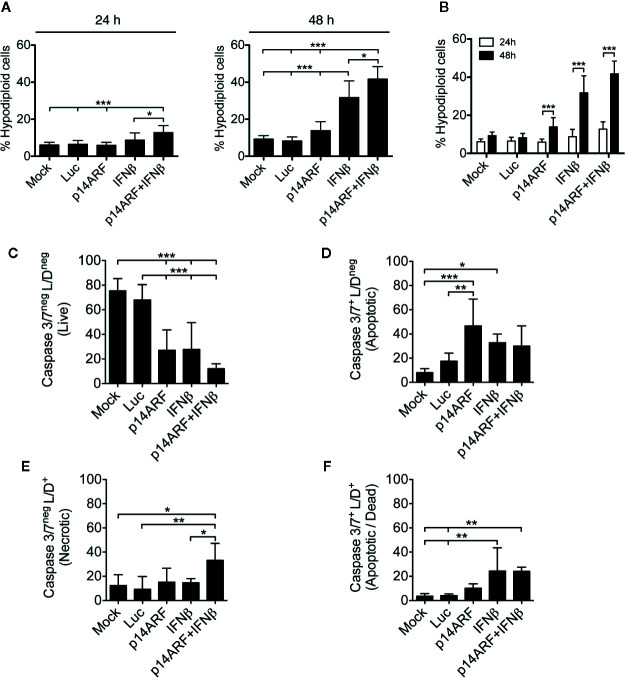
p14ARF and IFNβ gene transfer triggers cell death in human melanoma cells. SK-MEL-147 cells without treatment (Mock) or transduced with AdRGD-PG-Luc2 (Luc), AdRGD-Luc2-PG-p14ARF (p14ARF), AdRGD-PG-Luc2-IFNβ (IFNβ) or the combination (p14ARF + IFNβ) were subjected to cell death assays. **(A, B)** Graphs depict propidium iodide labeling performed 24 and 48 h after transduction. **(C–F)** Next, 48 h after transduction, cells were doubly marked for active caspase 3/7 and with LIVE/DEAD^®^ (L/D) to measure cellular membrane permeability. Thus, the following graphs demonstrate: **(C)** Live: Caspase 3/7^neg^, L/D^neg^, **(D)** Apoptotic: Caspase 3/7^+^, L/D^neg^, **(E)** Necrotic: Caspase 3/7^neg^, L/D^+^, and **(F)** Apoptotic/Dead: Caspase 3/7^+^, L/D^+^. Samples were acquired by flow cytometry. Mean and standard deviation were obtained from three independent assays each performed with technical triplicates. Statistical analyses were performed using one-way ANOVA test followed by Bonferroni post-test. *p < 0.05, **p < 0.005, and ***p < 0.0005.

Adenoviral vectors encoding Luc2, p14ARF or IFNβ were constructed (**Figure S1**) and transgene expression was validated (**Figures S2** and **S3**). In this work, we focus on the SK-MEL-147 human melanoma cell line since it harbors wild-type p53. Subsequently, we measured hypodiploid cell populations after transduction, revealing that the combination of adenoviruses p14ARF+IFNβwas superior to individual gene transfer for the induction of hypodiploidy (**Figure 1A**). **Figure 1B** shows the increase hypodiploidy over time, reaching 45% of cells in 48 h.

Next, we assessed the alteration of cell membrane permeability in a live/dead assay (L/D) and also measured caspase 3/7 activity 48 h after transduction. **Figure 1C** shows that the number of live cells (Caspase 3/7^neg^, L/D^neg^) was significantly reduced in the groups that received IFNβ, p14ARF or the combination as compared to the Mock or Luc control groups. Representative dot-blot graphs are shown in **Figure S4**. Apoptotic cells, those positive for caspase 3/7 activity (Casp3/7^+^) and with cell membrane integrity (L/D^neg^), were more frequent upon treatment with p14ARF (**Figure 1D**). In contrast, combined gene transfer was associated with necrosis (Casp3/7^neg^, L/D^+^), indicating loss of cell membrane integrity even without caspase 3/7 activity (**Figure 1E**). Caspase activity with concomitant loss of cell membrane integrity (Casp 3/7^+^, L/D^+^) was associated with IFNβ treatment alone or in combination with p14ARF (**Figure 1F**). These data indicate that treatment with p14ARF+IFNβ may induce cell death by a mechanism that is independent of caspase 3/7 activity.

### Nod-Scid Mouse Model of *In Situ* Gene Therapy Reveals Prolonged Survival in Response to Combined Gene Transfer

**Figure 2:**
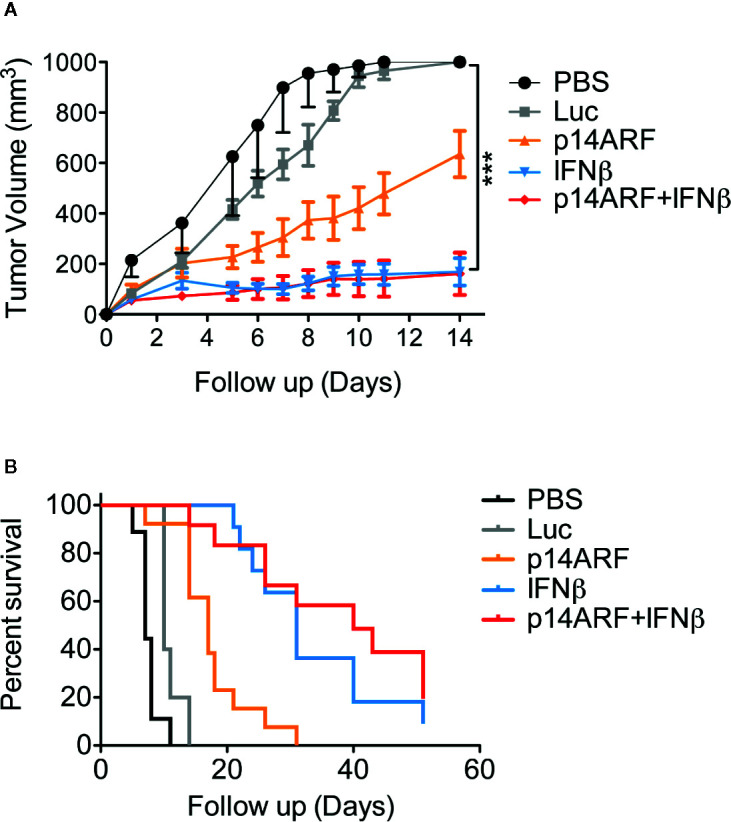
*In situ* gene therapy with the combination of p14ARF and IFNβ impaired tumor progression and improved survival rates in mouse xenograft model. Fresh SK-MEL-147 cells were inoculated in Nod-Scid mice, generating tumors after 7 days. The therapeutic regimen comprised four intra-tumoral injections at 48-h intervals with excipient alone (PBS, n = 9) or containing 2 × 10^8^ TU of AdRGD-PG-Luc2 (Luc, n = 5), AdRGD-PG-Luc2-p14ARF (p14ARF, n = 13), AdRGD-PG-Luc2-IFNβ (IFNβ, n = 11) or the combination (p14ARF + IFNβ, 1 × 10^8^ TU each virus, n = 12). **(A)** The tumor volume (mean +/- standard error) is shown in the graph. Statistical differences between IFNβ and p14ARF + IFNβ *versus* PBS and Luc were revealed on day 14 using unpaired t test with Welch’s correction. ***p < 0.0005. **(B)** Kaplan Meier graph shows survival (percent of mice with sub-maximum tumor size). Log-rank (Mantel-Cox) Test. p < 0.0001.

After seeing that adenovirus carrying the p14ARF and IFNβ transgenes triggered cell death in human melanoma cells *in vitro*, we questioned whether this potential could also be observed *in vivo*. For this, tumors were established by injection (s.c.) of 1 × 10^6^ cells in the right flank of Nod-Scid mice. Once the tumors had reached a volume of 60 mm^3^, we initiated the treatment regimen consisting of four doses of adenovirus (2 × 10^8^ TU per dose) administered by intratumoral injections with 48-h intervals between doses. The first day of treatment is considered as day 1 (thus virus was administered on days 1, 3, 5, and 7). The experimental endpoint in the PBS and Luc controls was reached around the tenth day of treatment, while IFNβ alone or in combination with p14ARF show significantly reduced tumor growth (**Figure 2A**) and prolonged survival (**Figure 2B**). We noticed an intermediate effect when p14ARF was used alone. While IFNβ was sufficient to inhibit tumor progression, we postulate that the benefit of p14ARF may lie in the mechanism of cell killing and the impact of oncolysis on the host response.

### Evidence of Immunogenic Cell Death Resulting From Adenoviral Treatments

**Figure 3:**
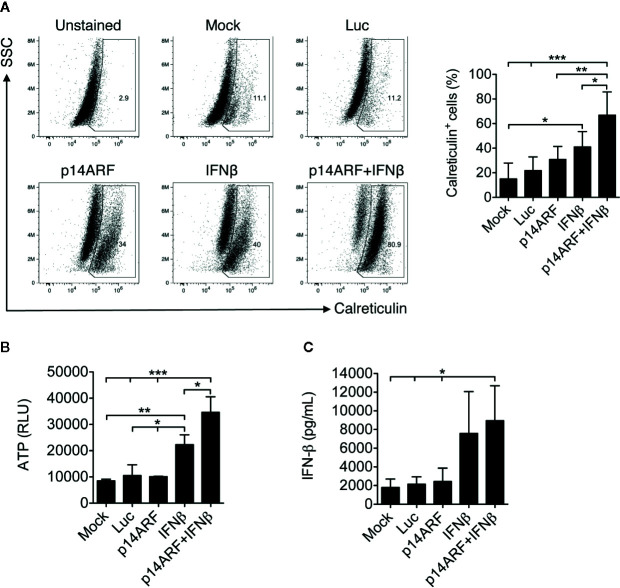
Emission of immunogenic cell death markers induced by combined adenoviral p14ARF + IFNβ gene transfer. SK-MEL-147 cells transduced as previously described, incubated for 48h h before cells and supernatants were collected for ICD assays. **(A)** Calreticulin exposure was assessed by flow cytometry after specific antibody staining. Representative dot plots and a bar graph showing the mean and standard deviation from three independent tests with three technical replicates each. **(B)** Supernatant from the same cultures were collected and evaluated for ATP secretion using a luciferase-based assay (RLU, relative light units). Data represent the mean and standard deviation from at least three independent experiments. **(C)** Detection of secreted IFNβ protein from cell supernatant by ELISA. Data represent the mean and standard deviation from at least three independent experiments. For both **(A–C)**, statistical analyses were performed using one-way ANOVA test followed by the Bonferroni post-test. *p < 0.05, **p < 0.005, and ***p < 0.0005.

Having verified the potential to generate oncolysis, we investigated whether treatment with the adenoviral vectors would trigger ICD, measured by the emission of DAMPs and activation of cellular mediators of the immune response. For this, SK-MEL-147 cells were transduced with the vectors encoding p14ARF, IFNβ or the combination and then incubated for 48 h before observing the exposure of calreticulin in the cell membrane and the secretion of ATP. **Figure 3A** shows that the increase in calreticulin exposure in the cell membrane of transduced cells was greatest in response to the IFNβ and p14ARF combination. This condition also resulted in increased granularity, indicating cellular stress. **Figure 3B** shows that secretion of ATP is most intensely induced by the p14ARF+IFNβ combination. The production of IFN-β, another DAMP, is also supported by our gene transfer approach (**Figure 3C**). Thus, combined gene transfer of p14ARF and IFNβ resulted in the emission of three critical DAMPs expected to contribute to immune cell activation.

### SK-MEL-147 Cells Transduced With p14ARF+IFNβ Adenoviral Vectors Induce Activation of Monocyte-Derived Dendritic Cells

**Figure 4:**
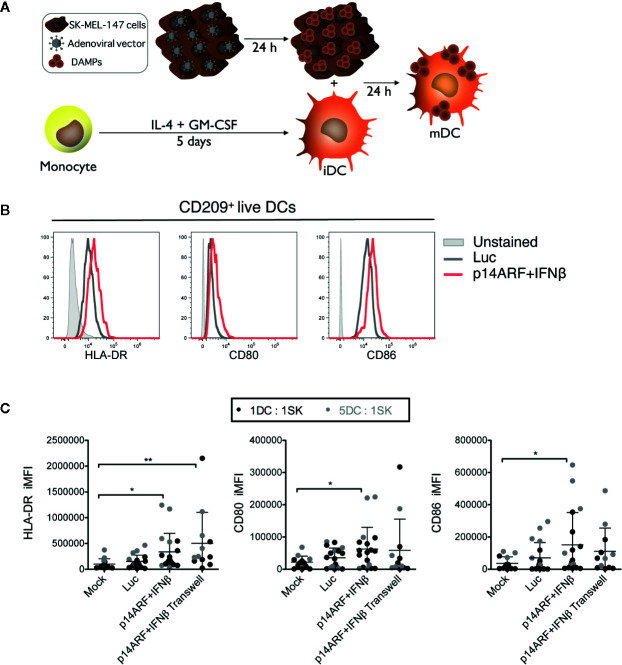
SK-MEL-147 cells transduced with p14ARF+IFNβ adenoviral vectors induced activation of monocyte-derived dendritic cells. **(A)** Schematic representation of the co-culture assay where SK-MEL-147 cells without treatment (Mock) or transduced with AdRGD-PG-Luc2 (Luc) or the combination AdRGD-Luc2-PG-p14ARF + AdRGD-PG-Luc2-IFNβ (p14ARF + IFNβ) were used to activate immature monocyte-derived dendritic cells (iDC). **(B)** Representative histograms and **(C)** frequency multiplied by MFI (iMFI) of HLA-DR, CD80 and CD86 in iDCs co-cultured in direct contact with transduced SK-MEL-147 cells or through a transwell membrane (0.4-μm pore size). Statistical analyses were performed using one-way ANOVA followed by the Bonferroni post-test. *p < 0.05, **p < 0.005.

To further investigate whether the induction of cell death and immunogenic factors upon transduction with the p14ARF+IFNβ adenoviral vectors could elicit an immune response, we exposed immature monocyte-derived dendritic cells (iDCs) to previously transduced SK-MEL-147 cells (**Figure 4A**). Combined p14ARF+IFNβ gene transfer was shown to be especially efficient for the release of ICD markers, thus was used in these assays. First, we verified that the viability of iDCs was not lost when co-cultured at different ratios (1:1 and 1:10) with SK-MEL-147 cells previously transduced at MOI 10, 50, or 100 (**Figure S5**). By gating on the live CD209^+^ iDCS, we are able to demonstrate that the frequency and expression level of HLA-DR, CD80, and CD86 increased in iDCs exposed to p14ARF+IFNβ-transduced SK-MEL-147 cells compared to iDCs exposed to mock-transduced SK-MEL-147 cells (representative histograms presented in **Figure 4B**). Statistically, iDCs behaved the same when treated at a ratio of 1:1 (black dots) or 5:1 (gray dots) iDC:SK-MEL-147 transduced cells, thus these results were pooled. When co-cultured in direct contact with iDC, p14ARF+IFNβ-transduced SK-MEL-147 cells induced an increase in expression of HLA-DR, CD80, and CD86 in iDCs, while co-culture in a transwell chamber revealed that soluble factors induced the upregulation of only HLA-DR in iDCs (**Figure 4C**). This suggests that membrane-associated factors presented by the transduced SK-MEL-147 cells are responsible for most of their dendritic cell activation effect.

### iDCs Activated With p14ARF+IFNβ-Transduced SK-MEL-147 Cells Modulate the Priming and Function of T Cells

**Figure 5:**
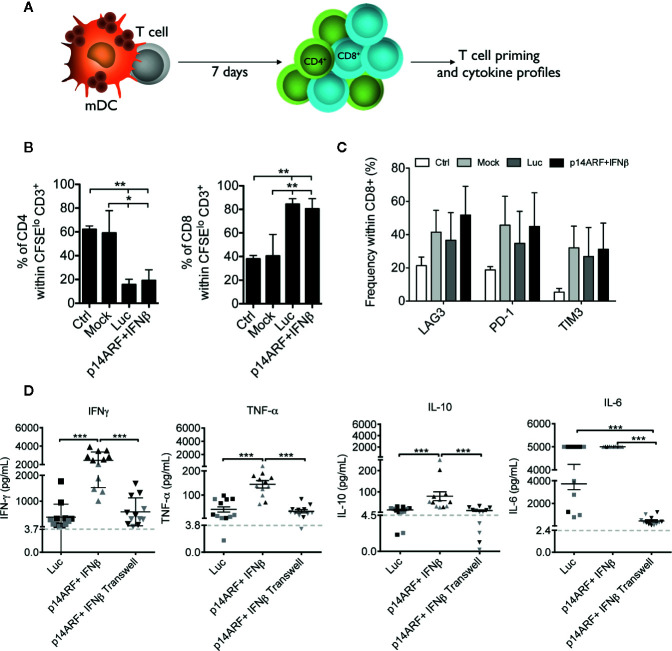
iDCs activated with p14ARF+IFNβ-transduced SK-MEL-147 cells modulate the priming and function of T cells. **(A)** Schematic representation of the DC-mediated priming of T cells. DCs (pre-activated with transduced SK-MEL-147 cells) were co-cultured with autologous T cells in the presence of IL-15, IL-7 and IL-2 to determine T cell proliferation and phenotypic profile. **(B)** Frequency of CD4^+^ and CD8^+^ T cells within CD3^+^ CFSE^lo^ (proliferating) T cells, primed by DCs previously activated with transduced SK-MEL-147 cells. Graphs show the mean and standard deviation from three independent assays. **(C)** Expression profile of checkpoint receptors in primed T cells. Graphs show the mean and standard deviation from three independent assays. **(D)** To determine the polarized cytokine profile, DCs were co-cultured with allogeneic T cells from two different donors (black and gray), to avoid the interference of external cytokines used in the autologous system. Cell-free supernatants were assessed for IFN-γ, TNF-α, IL-10, and IL-6 secretion by cytometric bead array. Gray line indicates the detection limit of each cytokine. Statistical analyses were performed using one-way ANOVA test followed by the Bonferroni post-test. *p < 0.05, **p < 0.005, and ***p < 0.0005.

Next, we explored whether iDCs exposed to transduced SK-MEL-147 cells were able to prime T cells and polarize cytokine production (**Figure 5A**). Using an autologous system supported by IL-15, IL-7, and IL-2, we found that iDCs activated with Luc and p14ARF+IFNβ-transduced SK-MEL-147 cells were able to switch the CD4/CD8 ratio, favoring the proliferation of CD8^+^ T cells rather than CD4^+^ (**Figure 5B**). Compared to unstimulated CD8^+^ T cells, iDCs activated with mock-transduced, Luc-transduced and p14ARF+IFNβ-transduced SK-MEL-147 cells favored a non-significant upregulation of the checkpoint molecules LAG-3, PD-1, and TIM-3 among the CD8^+^ T cell population (**Figure 5C**). To characterize the cytokine milieu induced during antigen presentation and co-stimulation, we co-cultured activated DCs with allogeneic T cells to avoid the interference of external cytokines needed in the autologous system. We found that iDCs activated with p14ARF+IFNβ-transduced SK-MEL-147 cells potently induced the production of IFN-γ by T cells, compared to iDCs activated with Luc-transduced SK-MEL-147 cells; TNF-α and IL-10 were also significantly increased, however at lower concentrations than IFN-γ. In turn, IL-6 was upregulated in T cells co-cultures with both, iDCs activated with Luc and p14ARF+IFNβ-transduced SK-MEL-147 cells. When iDCs were activated with p14ARF+IFNβ-transduced SK-MEL-147 cells separated through a transwell membrane, they lost the ability to induce cytokine production by T cells. No significant differences were found when iDCs were activated at a 1:1 (black) or 5:1 (gray) ratio with p14ARF+IFNβ-transduced SK-MEL-147 cells (**Figure 5D**).

### T Cells Primed by iDCs Activated With p14ARF+IFNβ-Transduced SK-MEL-147 Cells Kill Fresh Non-Transduced SK-MEL-147 Cells

**Figure 6:**
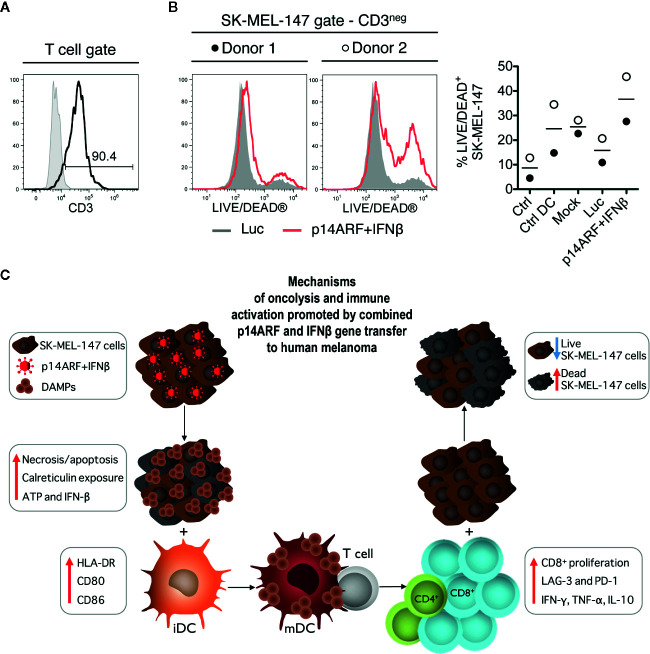
T cells primed by iDCs activated with p14ARF+IFNβ-transduced SK-MEL-147 cells induced viability loss of fresh non-transduced SK-MEL-147 cells. T cells primed with autologous DCs (previously activated with transduced SK-MEL-147 cells) from two healthy donors were challenged with fresh SK-MEL-147 cells to assess viability 48 h later. **(A)** Representative histogram of the frequency of CD3^+^ T cells, which comprised over 90% of the cell population among the lymphocyte gate (FSC^lo^). **(B)** Histograms showing the viability of SK-MEL-147 (CD3^neg^ FSC^hi^) after challenge with previously primed T cells. For both donors, there was an increase in dead cells triggered by the p14ARF + IFNβ combination group compared to the controls. **(C)** Schematic representation of the mechanisms of oncolysis and immune activation triggered by p14ARF + IFNβ gene therapy of melanoma cells.

Finally, to explore whether iDCs activated with adenovirus-transduced SK-MEL-147 cells were able to induce a specific cytotoxic population, autologous primed T cells from two healthy donors were challenged with fresh non-transduced SK-MEL-147 cells. After 48 h, we assessed the abundance of T cells in samples and the viability of CD3^neg^ cells within the SK-MEL-147 gate (higher FSC). We observed that within the lymphocyte gate, more than 90% of the cells were CD3^+^ (**Figure 6A**). For both donors, we found higher viability loss among fresh SK-MEL-147 cells when challenged with T cells primed by iDCs activated with p14ARF+IFNβ-transduced SK-MEL-147 cells, compared to all controls (**Figure 6B**). Based on these findings, we propose in **Figure 6C** the mechanism of immune activation in response to treatment of SK-MEL-147 cells with the combination of p14ARF+IFNβ gene transfer.

## Discussion

The classic conception of cancer therapy involves the use of chemotherapy and radiotherapy to induce cell death preferentially in tumor populations (44, 45). Frequently, these agents induce apoptosis, a well-known mechanism of cell death that involves the activation of caspases 3 and 7, fragmentation of DNA and packaging of cell content within portions of integral cell membrane (46–49). The maintenance of membrane integrity is an important feature of programmed cell death since this does not alarm the immune system (50). However, apoptosis may activate the proliferation of the remaining cells and thus reestablish the tumor mass (46, 51); therefore, alternative approaches that induce tumor cell death are needed.

In this paper, we constructed novel adenoviral vectors expressing p14ARF and IFNβ and used them individually or in combination for the transduction of SK-MEL-147 cells. Interestingly, our results show that the combination of p14ARF and IFNβ adenoviruses induced cell death independent of caspases3/7, unlike the IFNβ vector when used alone, corroborating other findings of our group when using mouse (21) or human cell lines (SM, OC, and BS, unpublished data).

The use of animal models to test *in situ* cancer gene therapy is an approach that allows us to make valuable inferences. Our group has accumulated evidence from previous studies that show adenoviral vectors, when injected intratumorally, are well tolerated by animals (20, 22, 52). Serum levels of ALT and AST did not show significant changes in a model of *in situ* gene therapy where only the IFNβ vector was administered, thus hepatotoxicity did not occur (53).

In this work, we investigated the potential antitumor effects of AdRGD-PG-IFNβ and AdRGD-PG-p14ARF in a model of *in situ* gene therapy using the human melanoma cell line SK-MEL-147 engrafted in Nod-Scid mice. Our results indicate that IFNβ gene transfer alone or when combined with p14ARF conferred potent inhibition of tumor progression, which directly reflects on the survival of these animals. In agreement with the *in vitro* cell death assays, p14ARF alone was less effective. The *in vivo* performance of our gene transfer approach corroborates with previous work (20, 22, 52), but nevertheless, it is unprecedented since this is the first demonstration of the use of a p53-responsive adenoviral vector for the transfer of p14ARF+IFNβ to human melanoma cells. Although the results were encouraging, the use of human cells in animal models brings an important limitation, the use of immunocompromised animals. Our previous data using the B16 cell line in immunocompetent mice pointed out that the contribution of the immune system to the control of tumors treated with the mouse transgenes (p19Arf + mIFNβ) is crucial (18, 20, 52).

One of our main motivations for the use of gene transfer to promote oncolysis was precisely the potential to modulate the tumor microenvironment, release tumor antigens, and DAMPs to stimulate the immune response. To this end, we investigated the occurrence of ICD. In this work, we focus on the exposure of calreticulin (54) and secretion of ATP (55) in response to gene transfer. Considered an early event of ICD, exposure of calreticulin on the cell surface acts as an “eat-me” signal for phagocytosis by macrophages, neutrophils, and DCs and is necessary for subsequent antigen cross-presentation to cytotoxic T cells (54, 56). Our data indicate that combined p14ARF+IFNβ gene transfer was the strongest inducer of ICD as measured by the release of DAMPS and motivated us to focus only on this group to continue the work. There are an increasing number of markers for characterizing ICD, including type I interferon (9, 10). Although we did not focus on expanding the number of ICD markers, we consider that the production of IFNβ encoded by the adenoviral vectors contribute to the ICD process.

Next, we characterized the immunogenic response itself, through assays with DCs and T cells. Our data suggest that the DAMPs and tumor antigens produced during the ICD of SK-MEL-147 cells upon transduction with p14ARF+IFNβ were able to activate iDCs, which in turn primed T cells to produce IFN-γ, TNF-α, and IL-10, and could also induce a specific cytotoxic population recognizing and killing SK-MEL-147 cells. In iDCs exposed to the factors induced by p14ARF+IFNβ, soluble factors, such as ATP and IFNβ, may be sufficient for the upregulation of HLA-DR, whereas calreticulin exposure and other contact-dependent factors are needed in addition to soluble factors to significantly increase the expression of the co-stimulatory molecules CD80 and CD86. Extracellular ATP released by dying cells guides the recruitment of antigen-presenting cells and promote debris uptake and clearance (54, 55, 57, 58) that in an IFN-β milieu, will increase the expression of interferon-stimulated genes, sustain MHC class II synthesis, antigen-processing and presenting capacity (59). The effect of calreticulin exposure, in turn, relies on the contact between iDCs and dying cells and the blockage of this pathway abolishes the immunogenicity of cell death (54, 60), perhaps by modulating the expression of co-stimulatory molecules in activated DCs as we observed in our transwell experiments. Indeed, the lack of contact between dying cells and iDCs (a.k.a. calreticulin-exposure signaling) also affects the subsequent polarization of T cells, which fail to improve their cytokine-producing capacity.

We explored antigen dosage by varying the number of transduced-SK-MEL-147 cells cultured with iDCs. While dosage had no significant impact on HLA-DR, CD80, and CD86 expression or the ability to induce cytokine-production by T cells, it appears that less antigen was better for the activation of DCs, particularly relevant when it comes to the induction of viable memory antigen-specific CD8^+^ T cells without exhausted phenotype (61, 62), that in our system remains to be investigated. Preliminary evidence obtained from four healthy donors show that iDCs activated with both Luc and p14ARF+IFNβ-transduced SK-MEL-147 cells were biased to induce the proliferation of CD8^+^ T cells, due to the adenoviral-transduction itself and not necessarily by p14ARF+IFNβ. However, only p14ARF+IFNβ was able to polarize the cytokine milieu toward a Th1 profile, suggesting that our combination could effectively trigger a desirable cytokine setting for an antitumor response (63).

The upregulation of checkpoint molecules in CD8^+^ T cells has been proposed to distinguish tumor-specific clones that had recent TCR activation (64). The transient invigoration of exhausted CD8^+^ T cells to potent tumor-reactive cells has been achieved through the combination of anti-PD-1 therapy with PEGylated IL-10, leading to the expansion of a rare population of LAG-3^+^ PD-1^+^ CD8^+^ T cells that positively correlates with clinical response (65). Intriguingly, iDCs activated with p14ARF+IFNβ-transduced SK-MEL-147 cells favored the upregulation of LAG-3 and PD-1 in CD8^+^ T cells simultaneously with IL-10 production; the significance of this phenomena to our strategy is unknown but noteworthy since one of the biggest challenges of modern immunotherapy strategies is the induction of potent antitumor response without the adverse events arising from an uncontrolled immune response. Future studies of our strategy will address the kinetics of cytotoxic and exhausted CD8^+^ T cell populations and the significance of the simultaneous checkpoint molecule and IL-10 upregulation.

Beyond the phenotype and function of the CD209^+^ monocyte-derived DCs characterized here, DAMPs and ICD triggered by p14ARF+IFNβ could also influence other circulating DC populations potentially present in the adherent and non-adherent fractions, sources of monocytes and T cells. Of note, plasmacytoid DCs (pDC) with its remarkable antiviral capacity and production of type I interferons, slanDCs the major source of IL-12 that favor Th1 responses and cDC1 CD141^+^, specialists in antigen cross-presentation to CD8^+^ T cells (66, 67). Each subpopulation, that substantially differ in terms of function, might also have important differences in their capacity to internalize calreticulin-exposing dying cells, the repertoire of pattern recognition receptors (PRRs) recognizing p14ARF+IFNβ-induced DAMPs, as well as signaling toward inflammatory and anti-inflammatory cytokines and ability to polarize adaptive responses (68, 69). Also, by our methods, we could not rule out the participation of other immune cells, such as NK cells; previous *in vivo* work from our group points to the participation of NK cells in our strategy and poorer antitumor effect after *in situ* IFNβ-vector treatment of SK-MEL-147 tumors in Nod-Scid mice *versus* nude (OC and BS, manuscript submitted). In a mouse model, we have observed activation of ULBP1, IL-15, Killer/DR5 and Fas/Apo1 in B16 tumor cells specifically in response to combined p19Arf+mIFNβ gene transfer (52). Additional studies are needed to understand the participation of other lymphocyte populations, such as CD4^+^ helper T cells and NK cells in the mechanisms described here.

In summary, we present evidence that p14ARF+IFNβ-transduction of human melanoma cells leads to ICD that activates iDCs, which in turn, induce and polarize the adaptive immune response toward a Th1 profile. Data from our experiments with T cells suggest that the expansion of antigen-specific CD8^+^ T cells would be expected to participate in controlling tumor progression *in vivo*. Based on our *in vitro* proof of concept data, further exploration of immune cell activation using healthy donors or, ideally, from melanoma patients is warranted.

## Data Availability Statement

The raw data supporting the conclusions of this article will be made available by the authors, without undue reservation.

## Ethics Statement

The studies involving human participants were reviewed and approved by Comite de Etica em Pesquisa, Pró-Sangue, Faculdade de Medicina, Universidade de Sao Paulo. Written informed consent for participation was obtained for this study in accordance with the national legislation and the institutional requirements. The animal study was reviewed and approved by Comite de Etica em Uso de Animais (CEUA), Faculdade de Medicina, Universidade de Sao Paulo.

## Author Contributions

OC: experimental design, coordinated assays, *in situ* therapy in animal models, *in vitro* co-culture of tumor and immune cells experiments, western-blot, immunofluorescence, microscopy, data analysis, and manuscript writing. MC-S: design of immunological assays, co-culture of tumor and immune cells experiments, ELISA, flow cytometry, data analysis, and manuscript writing. EC: co-culture of tumor and immune cells experiments, CBA, flow cytometry, data analysis, and manuscript writing. TC: hypodiploid assays, flow cytometry analysis, and manuscript writing. SM: adenovirus construction and production. JB: design of immunological assays and data interpretation. BS: coordinated supporting grants, experimental design, data interpretation, manuscript writing and editing. All authors contributed to the article and approved the submitted version.

## Funding

This work was supported by the Sao Paulo Research Foundation (FAPESP) grant 2015/26580-9 (BS), post-doctoral fellowships 2017/23068-0 (OC) and 2017/13686-9 (MC-S), and Conselho Nacional de Desenvolvimento Científico e Tecnológico (CNPq) fellowship 302888/2017-9 (BS).

## Conflict of Interest

The authors declare that the research was conducted in the absence of any commercial or financial relationships that could be construed as a potential conflict of interest.
