# Supplementary material for: Combined p14ARF and Interferon-β Gene Transfer to the Human Melanoma Cell Line SK-MEL-147 Promotes Oncolysis and Immune Activation
Source: Front Immunol. 2020 Oct 22;11:576658. doi: 10.3389/fimmu.2020.576658 (PMC7642851; doi:10.3389/fimmu.2020.576658)
Supplement: Supplementary file 1 [file DataSheet_1.pdf]

Supplemental Data

Combined p14ARF and interferon-β gene transfer to the human melanoma cell line SK-MEL-147 promotes oncolysis and immune activation

Otto Luiz Dutra Cerqueira, PhD[1*]; Maria Alejandra Clavijo-Salomon, PhD[1,2*]; Elaine Cristina Cardoso, PhD[3], Tharcisio Citrangulo Tortelli Junior, PhD[1], Samir Andrade Mendonça, PhD[1,4], José Alexandre M. Barbuto, PhD [2,5], Bryan E. Strauss, PhD[1,6]

*These authors contributed equally to this work

[1]Centro de Investigação Translacional em Oncologia (CTO), Instituto do Câncer do Estado de São Paulo (ICESP), Faculdade de Medicina da Universidade de São Paulo (FMUSP), São Paulo, SP, 01246-000, Brazil

[2]Departamento de Imunologia, Instituto de Ciências Biomédicas, Universidade de São Paulo, São Paulo, SP, 05508-000, Brazil

[3]Departament of Pediatrics, Faculdade de Medicina da Universidade de São Paulo (FMUSP), São Paulo, SP, 01246-000, Brazil

[4]Department of Radiation Oncology, Washington University School of Medicine in St. Louis, MO,United States

[5]Laboratory of Medical Investigation in Pathogenesis and Targeted Therapy in Onco-Immuno-Hematology (LIM-31), Department of Hematology, Hospital das Clínicas HCFMUSP, Faculdade de Medicina, Universidade de Sao Paulo, Sao Paulo, Brazil.

[6]Corresponding author: Bryan E. Strauss, Ph.D.

Centro de Investigação Translacional em Oncologia-(CTO), ICESP, Av. Dr. Arnaldo 251, 8th floor, Cerqueira César, São Paulo, SP, 01246-000, Brazil

Tel: +55(11)3893-3554

bstrauss@usp.br; bryan.strauss@hc.fm.usp.br

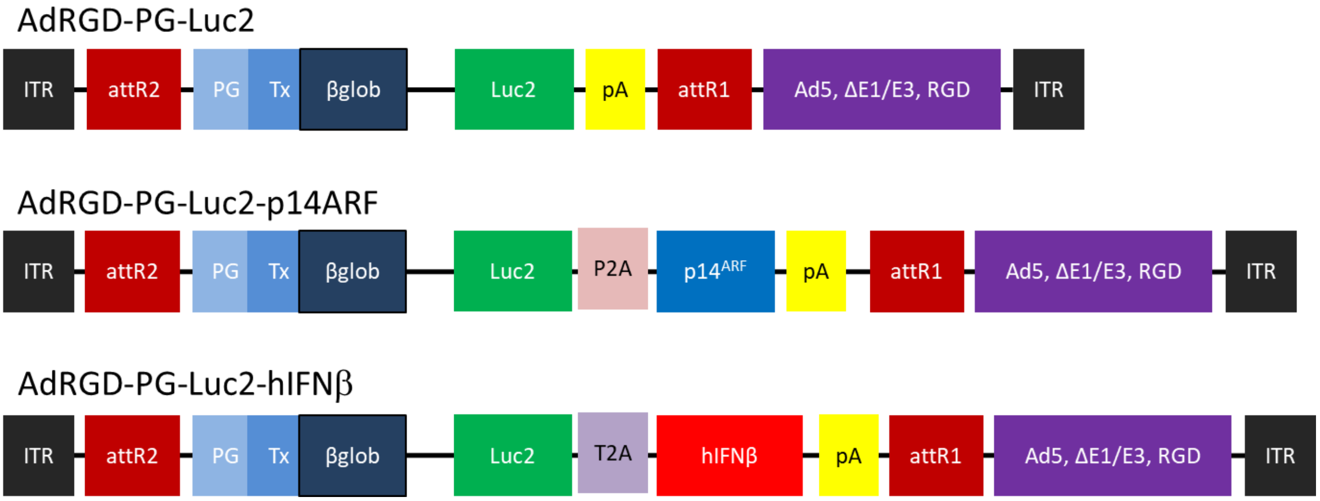

Figure S1. Schematic representation of adenoviral vectors (AdRGD-PG). ITR: inverted terminal repeats; attR1 and attR2: site-specific recombination sequences; PG/Tx/βglob: p53-responsive promoter, composed of the PG enhancer sequence, TATA box (Tx) and βglobin (βglob) intronic sequences; Luc2: Luciferase 2; P2A: 2A self-cleaving peptides; T2A: T2A self-cleaving peptides; p14ARF: Alternative reading frame (CDKN2a) cDNA sequence; IFNβ: human interferon-β cDNA sequence; pA: polyadenylation sequence; Ad5, ΔE1/E3: Adenovirus 5 backbone with deletion of genes E1 and E3; RGD, Arginine-glycine-aspartic acid tripeptide modification of the knob protein.

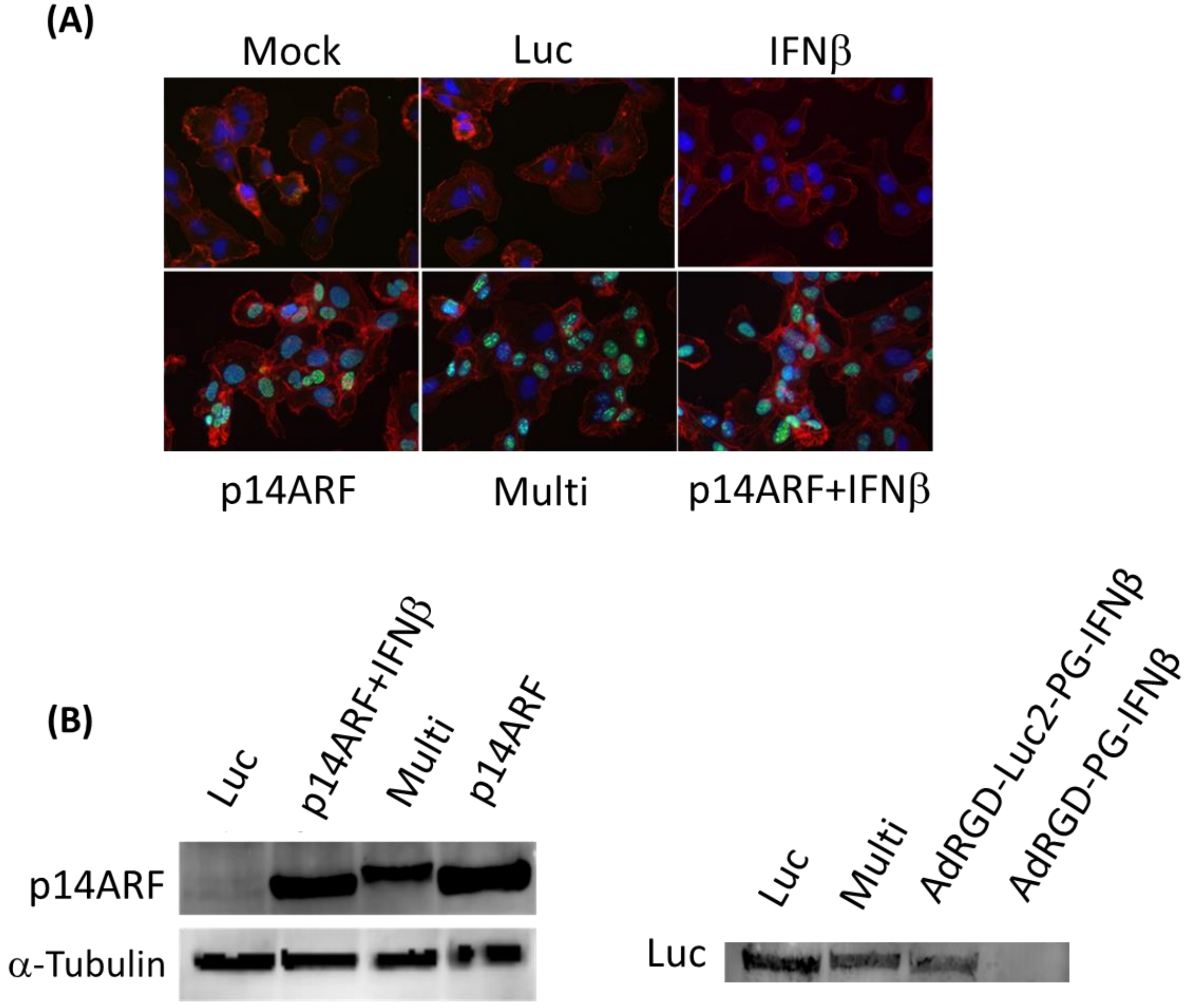

Figure S2. Validation of transgene expression. SK-MEL-147 cells were transduced (MOI 50) with AdRGD-PG-Luc2 (Luc), AdRGD-Luc2-PG-p14ARF (p14ARF), AdRGD-PG-Luc2-IFNβ (IFNβ), the combination (p14ARF + IFNβ), or AdRGD-Luc2-PG-p14ARF-IFNβ (multi) or the AdRGD-PG-IFNβ vector which does not encode Luc2, then cells were incubated for 48 hours. **A**) Immunofluorescence detection of p14ARF in SK-MEL-147 cells transduced with adenoviral vectors. p14ARF (green), phalloidin (red) and DAPI (blue) were detected upon fluorescence microscopy (EVOS FL, Thermo Fisher Scientific, Waltham, MA, USA) using a 20x objective lens. **B)** Western blot for detection of Luc and p14ARF proteins in SK-MEL-47 cells. Alpha-Tubulin was used as a loading control.

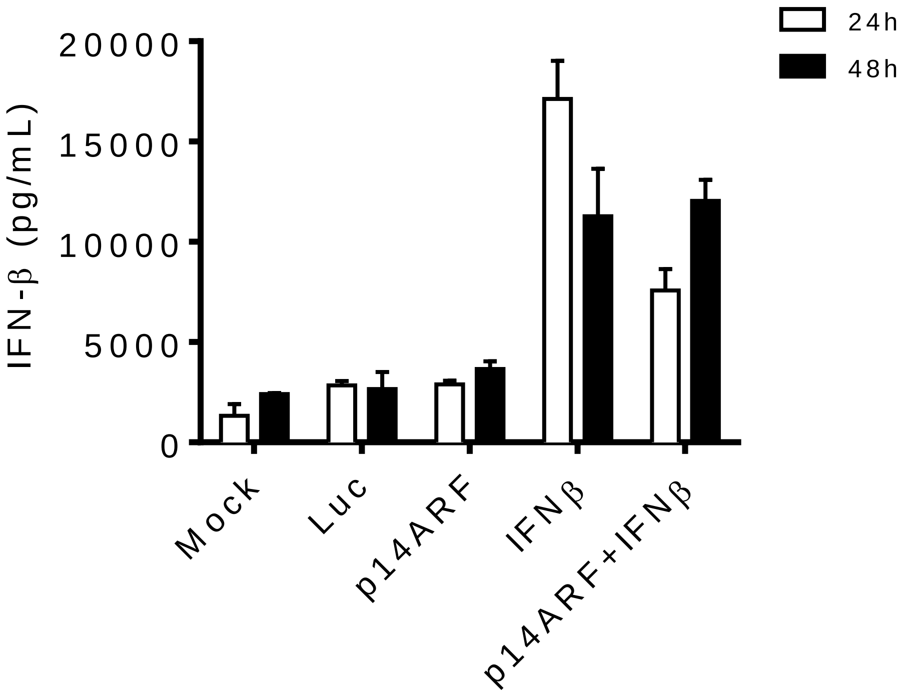

Figure S3. Validation of IFNβ expression. ELISA for human IFNβ performed on supernatants from SK-MEL-147 cultures transduced with the respective adenoviral vectors (MOI 50).

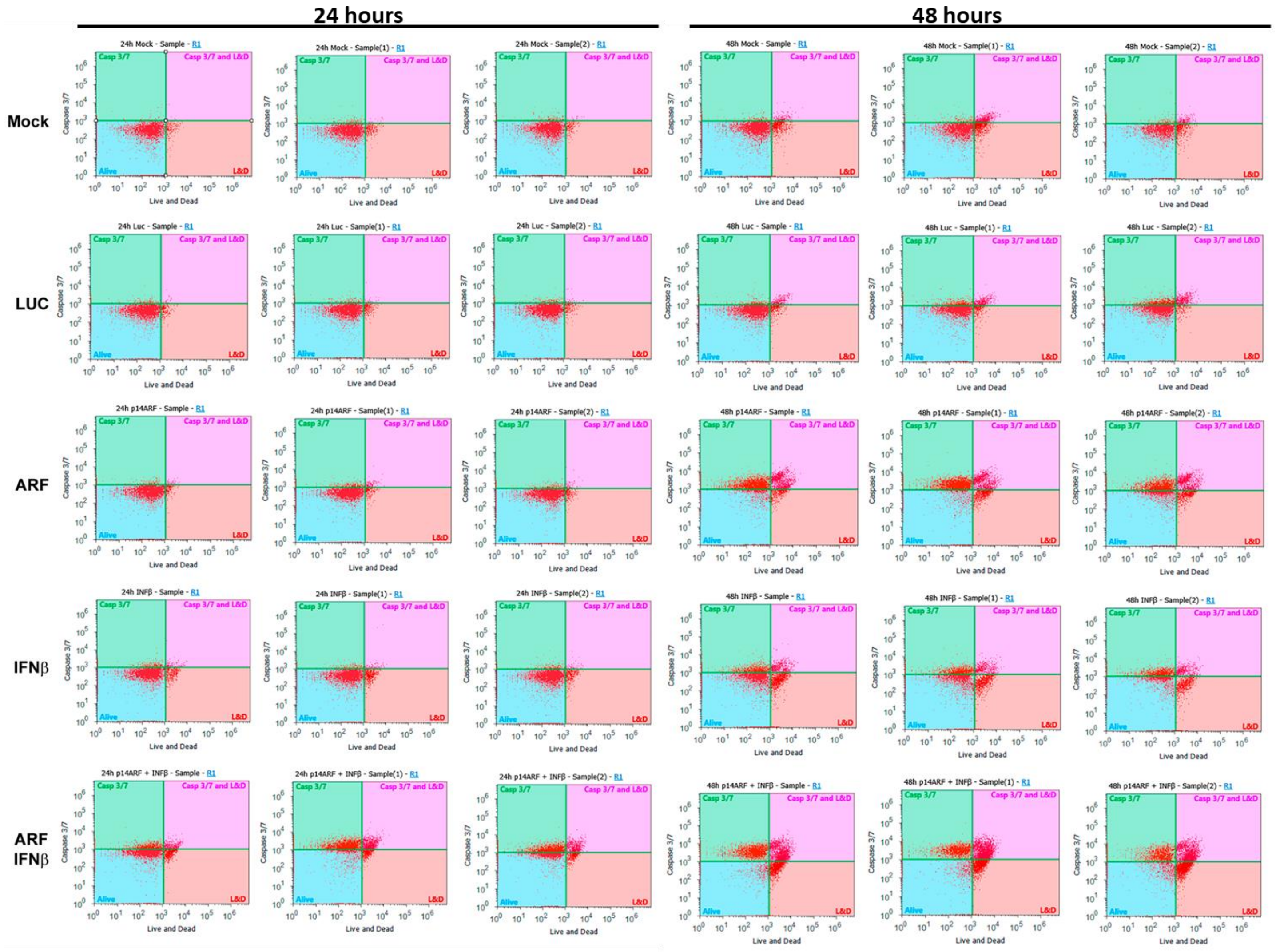

Figure S4. Representative images of flow cytometry dot plots demonstrating caspase 3/7 activity and Live/Dead (L/D) assays. Data used to derive Figures 1C, D, E and F**.** For each condition, three technical replicates were collected 24 and 48 hours after adenoviral transduction.

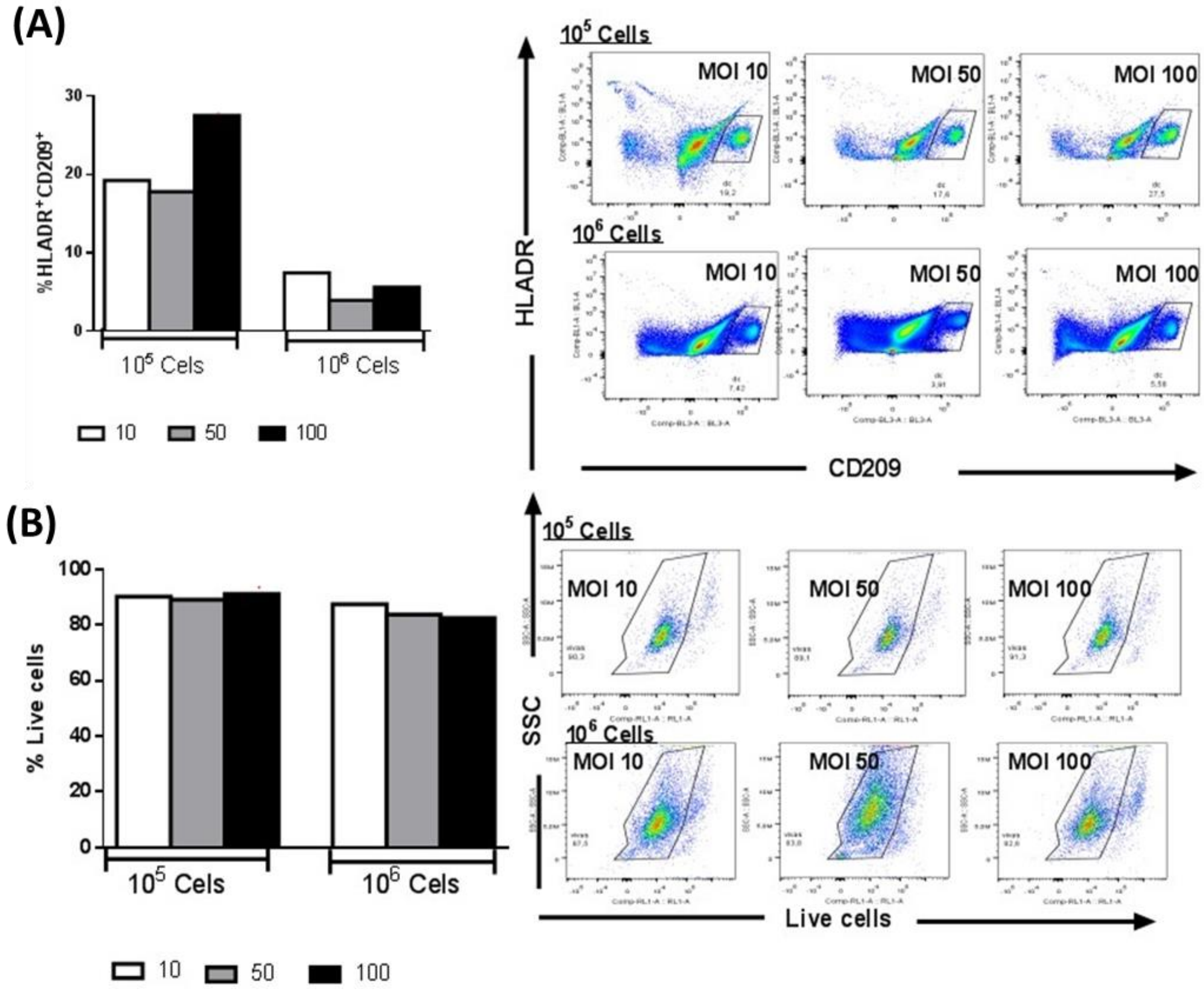

Figure S5. DC viability after co-culture with transduced SK-MEL-147 cells. In preparation for the *ex vivo* activation of DCs, we initially standardized different MOI's (10, 50, 100), as well as the proportion of transduced tumor cells for each DC (10^5: 1 tumor cell for 1 DC and 10^6: 10 tumor cells for 1 DC). A) Gate strategy for identifying DCs: HLA-DR$^+$ vs. CD209$^+$. B) Evaluation of cell viability of the DC subpopulation.
